# Physiological Mechanisms for High Salt Tolerance in Wild Soybean (*Glycine soja*) from Yellow River Delta, China: Photosynthesis, Osmotic Regulation, Ion Flux and antioxidant Capacity

**DOI:** 10.1371/journal.pone.0083227

**Published:** 2013-12-12

**Authors:** Peng Chen, Kun Yan, Hongbo Shao, Shijie Zhao

**Affiliations:** 1 Key Laboratory of Coastal Biology & Bioresources Utilization and Key Laboratory of Coastal Zone Environmental Processes, Yantai Institute of Coastal Zone Research (YIC), Chinese Academy of Sciences (CAS), Yantai, China; 2 Institute for Life Sciences, Qingdao University of Science & Technology (QUST), Qingdao, China; 3 State Key Laboratory of Crop Biology, Shandong Agriculture University, Tai’an, China; Universidade Federal do Rio de Janeiro, Brazil

## Abstract

*Glycine soja* (BB52) is a wild soybean cultivar grown in coastal saline land in Yellow River Delta, China. In order to reveal the physiological mechanisms adapting to salinity, we examined photosynthesis, ion flux, antioxidant system and water status in *Glycine soja* under NaCl treatments, taking a cultivated soybean, ZH13, as control. Upon NaCl exposure, higher relative water content and water potential were maintained in the leaf of BB52 than ZH13, which might depend on the more accumulation of osmotic substances such as glycinebetaine and proline. Compared with ZH13, activities of antioxidant enzymes including superoxide dismutase, catalase, ascorbate peroxidase and contents of ascorbate, glutathione and phenolics were enhanced to a higher level in BB52 leaf under NaCl stress, which could mitigate the salt-induced oxidative damage in BB52. Consistently, lipid peroxidation indicated by malondialdehyde content was lower in BB52 leaf. Photosynthetic rate (Pn) was decreased by NaCl stress in BB52 and ZH13, and the decrease was greater in ZH13. The decreased Pn in BB52 was mainly due to stomatal limitation. The inhibited activation of rubisco enzyme in ZH13 due to the decrease of rubisco activase content became an important limiting factor of Pn, when NaCl concentration increased to 200 mM. Rubisco activase in BB52 was not affected by NaCl stress. Less negative impact in BB52 derived from lower contents of Na^+^ and Cl^-^ in the tissues, and non-invasive micro-test technique revealed that BB52 roots had higher ability to extrude Na^+^ and Cl^-^. Wild soybean is a valuable genetic resource, and our study may provide a reference for molecular biologist to improve the salt tolerance of cultivated soybean in face of farmland salinity.

## Introduction

At present, more than 800 million hectares of lands are affected by salinity in the world and in particular, farmland salinity has become a severe agricultural problem due to unreasonable irrigation and fertilization [1,2]. Soil salinity inhibits plant growth and decreases crop yield, and it is a feasible way to meet the challenge of farmland salinity by enhancing crop salt tolerance [3]. Halophyte can survive and evolve in saline lands because of some special physiological adaptive mechanisms [4], and it is a primary work to explore these adaptive mechanisms before gene transformation for improving salt tolerance of cultivated crop species. In general, negative effects of salt stress on plants are ascribed to osmotic stress, ion toxicity and oxidative stress [1,5]. Upon salinity exposure, osmotic stress often firstly occurs because water absorption by plant roots becomes hard, and many osmotic regulation substances may be accumulated to increase their hyperosmotic tolerance for avoiding water loss from the cells. Proline and glycinebetaine are the most widely reported osmotic regulation substances, and their important role in resisting salt stress also has been recognized [6]. In order to reduce water loss under saline condition, plants can down regulate leaf stomatal conductance to reduce water evaporation, but photosynthesis is concomitantly affected, as CO_2_ flux into leaf is also inhibited. Photosynthesis is closely related with plant growth and crop yield and is also very sensitive to environmental stress. Depression of photosynthetic rate can be usually found in plants under salt stress, but the underlying reasons remain to be ascertained [7-9]. Initial negative effect of salt stress on photosynthesis may be due to the decreased CO_2_ availability as a result of the diffusion limitations of stomata [7,10]. Rubisco is the key enzyme in CO_2_ fixation process, and rubisco activity is liable to be suppressed under salt stress, however, decreased rubisco activity stems from different aspects such as decreased rubisco content and inhibited rubisco activation [8,11]. Photosystem II (PSII) plays an important role in plant photosynthesis, but its tolerance to salt stress still remains to be defined. PSII was proved to be sensitive to salt stress in some studies [9,12,13], whereas high salt-resistance of PSII was demonstrated in other studies [10,14,15].

Salt stress can bring about oxidative stress on plants by inducing excess generation of reactive oxygen species (ROS) in plant tissues [16]. Antioxidant system including antioxidant enzymes such as superoxide dismutase (SOD) and ascorbate peroxidase (APX), and antioxidants such as ascorbic acid (AsA) and glutathione (GSH) to scavenge excess ROS, and the antioxidant capacity are often used for differentiating the salt tolerance in crop varieties [17,18]. Na^+^ and Cl^-^ are the origins exerting toxic effects on physiological metabolisms and plant growth in saline condition. Except the succulent species such as *Suaeda salsa*, salt-tolerant plants often have high extraction capacity of Na^+^ and Cl^-^ in roots, and then maintain lower Na^+^ and Cl^-^ in the tissue. Sun et al. [19] reported that salt-tolerant *Populus euphratica* exhibited a higher capacity to extrude Na^+^ than salt-sensitive *Populus popularis* under NaCl stress, and Na^+^/H^+^ antiporter played a crucial role in ion homeostasis. Luo et al. [20] reported that *G. soja* had higher salt tolerance than *G. max* due to less accumulation of Na^+^ and Cl^-^. Zhang et al. [21] revealed that *G. soja* had higher Na^+^ and Cl^-^ extrusion in roots in contrast to *G. max*.

Soybean is rich in proteins and necessary for the diet of Human beings. There is a great of demand for soybean in the international market. However, cultivated soybean species are usually sensitive to soil salinity, and farmland salinity can lead to the loss of soybean yield. *Glycine soja* (BB52) is a wild soybean cultivar grown in coastal saline land in Yellow River Delta, China. *G. soja* is the near relative ancestor of cultivated soybean species, and they have high gene homology [22,23]. In addition, as a salt-born soybean species, *G. soja* should have unique physiological mechanisms for adapting to salinity stress in contrast to cultivated soybean. Therefore, *G. soja* can be considered as a valuable genomic resource for improving salt tolerance of cultivated soybean species by molecular biology means. Up to now, it has been reported about the salt tolerance in *G. soja* with higher root extrusion and less accumulation in tissues of Na^+^ and Cl^-^ [21], however, the systematic salt-adaption physiological mechanisms have not been comprehensively studied in *G. soja*. In this study, we intended to systematically diagnose these mechanisms in *G. soja* from the aspects of root ions flux, antioxidant system, osmotic regulation and photosynthesis, and the results can enrich the knowledge of plant salt tolerance and may provide a reference for genetic improvement of salt tolerance in cultivated soybean.

## Materials and Methods

"I state clearly that no specific permissions were required for these locations/activities, which are concerned about scientific study in the region in China. I confirm that the field studies did not involve endangered or protected species".

### Plant material and treatment

*G. soja* (BB52) is a wild soybean cultivar grown in coastal saline land in Yellow River Delta. The area belongs to temperate humid continental monsoon climate. *G. max* (ZH13) is a widely planted soybean cultivar in China, and it has high yield. The seeds of BB52 were collected in Yellow River Delta in October, 2012, while the seeds of ZH13 were obtained from Shandong Academy of Agricultural Science. The seeds of ZH13 were fully soaked in distilled water for 8 h, while the seeds of BB52 were soaked in concentrated sulfuric acid for 10 min to remove the hard shell over the seeds. Then, the seeds were placed in petri dishes in the dark between two sheets of filter paper at 25 °C to germinate, and the filter paper was kept wet by spraying Hoagland nutrient solution (pH 5.7). After 5 days, the germinated seeds were transferred to plastic pots filled with vermiculite and grown in artificial climatic chambers (Huier, China). The vermiculite was kept wet by watering also with Hoagland nutrient solution every day. Light time was 12 hours in each day. Day/night temperature and humidity were respectively controlled at 25/18 °C and 65% in the chambers. After 20 days, seedlings with uniform growth pattern were selected for salt treatment. NaCl was added to nutrient solution to provide final concentrations of 0 (control), 50, 100, 200 and 300 mM. The higher NaCl concentrations (>50 mM) were imposed incrementally by 50 mM step every day until final concentrations were reached. There were twenty replicate seedlings in each salt treatment, and each parameter was measured for five times respectively by using five replicate seedlings 7 days after the final concentrations were reached. Photosynthetic, fluorescent, water potential and relative water content measurements were conducted on the newest fully expanded leaves, and this kind of leaves were sampled, frozen in liquid nitrogen and stored at -80 °C in a freezer for measuring other parameters such as antioxidant, osmoregulation substance and malondialdehyde (MDA) contents, antioxidant enzymes and rubisco activities, and western blotting. The roots and leaves (the newest fully expanded) were harvested from five replicate seedlings and dried at 70 °C for 48 h. The dried samples were ground to powder and used for measuring Na^+^ and Cl^-^ contents.

### Measurements of gas exchange and modulated chlorophyll fluorescence

Gas exchange and modulated chlorophyll fluorescence were simultaneously detected using an open photosynthetic system (LI-6400XT, Li-Cor, USA) equipped with a fluorescence leaf chamber (6400-40 LCF, Li-Cor, USA). The leaves were dark-adapted for 30 min before the measurements. The minimal fluorescence level in the dark-adapted state (Fo) was measured using a modulated pulse (< 0.05 µmol photons m^-2^ s^-1^ for 1.8 s). Maximal fluorescence (Fm) was measured after applying a saturating actinic light pulse of 8000 µmol photons m^-2^ s^-1^ for 0.7 s. Subsequently, actinic light intensity was altered to 1000 µmol photons m^-2^ s^-1^ in leaf cuvette and then maintained for about 30 min. The temperature, carbon dioxide concentration and relative humidity in the leaf cuvette depended on ambient conditions. Stomatal conductance (Gs), intercellular CO_2_ concentration (Ci) and transpiration rate (E) were recorded simultaneously with Pn. In addition, steady-state fluorescence yield (Fs) was also recorded. A saturating actinic light pulse of 8000 µmol photons m^-2^ s^-1^ for 0.7 s was then used to produce maximum fluorescence yield (Fm’) by temporarily inhibiting photosystem II (PSII) photochemistry, and then the actual photochemical efficiency of PSII (ΦPSII) were calculated [24].

For the measurement of carboxylation efficiency (CE), photon flux density and temperature were set at 1000 µmol photons m^-2^ s^-1^ and 25 °C in the leaf cuvette, respectively. Pn was measured under CO_2_ concentrations in a sequence of 600, 500, 400, 300, 200, 150, 100, and 50 µmol mol^-1^. The leaves were kept under each level of CO_2_ concentration for 5 min to let leaves reach steady photosynthesis, the Pn and Ci were then recorded. The correlation curve of Pn related to Ci was established. CE was calculated from the linear portion of the Pn-Ci curve according to Chen et al. [25].

### Measurement of chlorophyll content

Leaf samples (0.2 g) were soaked in 20 ml 95% (v/v) ethanol at 4 °C in darkness until the tissues became totally white. Extracts were used to measure the absorbance at 649 nm and 665 nm, the chlorophyll content was calculated according to Lichtenthaler and Wellburn [26].

### Measurement of rubisco activity and activation state

Rubisco activity was measured according to Wang et al. [27] with slight modifications. Leaf samples (0.2 g) were frozen in liquid nitrogen and homogenized to fine powder with mortar and pestle. Rubisco was extracted by grinding the fine powder in 1 ml extraction buffer containing 100 mM HEPES-KOH (pH 8.0), 10 mM MgCl_2_, 0.5 mM ethylene diaminetetraacetic acid (EDTA), 1% (w/v) polyvinylpolypyrrolidone (PVPP) and 0.06 ml b-mercaptoethanol. After centrifugation at 16000 g for 15 min, the supernatant was used in the measurement of initial activity of rubisco. Extract supernatant (0.1 ml) was added to 0.4 ml of activation solution (33 mM HEPES-KOH (pH 8.0), 33 mM MgCl_2_, 0.67 mM EDTA, 10 mM NaHCO_3_ as final concentration). After incubated at 30 °C for 10 min, the solution was used for measurement of total activity of rubisco.

The activity of rubisco was determined spectrophotometrically by measuring the disappearance rate of NADH. To determine the initial and total activity of rubisco, the reaction was initiated by adding 60 µl 10 mM ribulose-1,5-diphosphate (RuDP) and 0.1 ml extract immediately after mixing the desalted sample solution containing 50 mM HEPES-KOH (pH 8.0), 20 mM MgCl_2_, 1 mM EDTA, 2.5 mM DTT, 2.5 mM NADH, 5 mM ATP, 10 mM NaHCO_3_, 5 mM phosphocreatin, 10 U/ml of phosphocreatine kinase, 10 U/ml of phosphoglyceric kinase and 10 U/ml of glyceraldehyde-3-phosphate dehydrogenase. The changes in the absorption were recorded and the activation state of rubisco was calculated as the ratio of initial activity to total activity of this enzyme.

### Western blotting of rubiscoactivase

Leaf samples (0.5 g) were ground in liquid nitrogen with mortar and pestle. Total proteins were firstly extracted with 1 ml buffer I (acetone containing 10% (W/V) TCA and 0.07% (V/V) b-mercaptoethanol). After incubated at -20 °C for 1 h, the mixture was centrifugated for 30 min (4 °C, 15000 g). Equal volume of buffer II (acetone containing 0.07% (V/V) b-mercaptoethanol) was added and the mixture was incubated and centrifugated as above after vortexing for 1min. Repeat this step twice to remove salt ion and organic admixture like pigmentum and polyphenol. The protein pellets were vacuum-dried, recovered in lysis buffer and centrifugated for 30 min (4 °C, 15000 g). The upper phase protein concentrations were quantified using the Bradford assay [28].

For western-blotting, the same quantityprotein of BB52 and ZH13 was separated by SDS-PAGE using 12% (w/v) acrylamide gels and electrically transferred onto polyvinylidene fluoride (PVDF) membranes (Clontech, China) using a semi-dry transfer system (BIO-RAD, USA). The protein blot was probed with a primary antibody of the rubisco activase (Agrisera, Sweden, dilution of 1:5000) for 2 h at 37 °C with agitation and then incubated with the secondary antibody (peroxidase-babeled affinity purified antibody, KPL, USA, dilution of 1:1000) for 2 h at 37 °C. The blots were finally developed with a peroxidase substrate kit (KPL, USA).

### Measurement of water potential (ψ_w_) and relative water content (RWC)

Leaf samples were placed in a sample cup, and the cup bottom should be completely covered. Then, water potential was measured using chilled-mirror dewpoint technique with a WP4-T Dewpiont Potentia Meter (USA). Fresh leaves were harvested and weighed (fresh weight, FW), and then soaked in deionized water for 24 h at 4 °C and weighed (called saturated fresh weight, SFW). Finally, the leaves were dried completely in an oven and weighed (dry weight, DW). RWC was calculated as: RWC= (FW-DW) / (SFW-DW).

### Measurement of glycinebetaine content

Leaf samples (0.2 g) were ground under liquid nitrogen and homogenized in 3 ml methanol-chloroform-KHCO_3_ solution (methanol: chloroform: 0.2 mM KHCO_3_= 12:5:1). The mixture was incubated at 60 °C in water bath for 20 min and then centrifugated at 10000 g for 10 min. The supernatant was transferred to another tube and the precipitate was washed twice (firstly with 1 ml of the same extract solution, secondly with 1 ml of methanol- H_2_O (1:1) solution, and all transferred to the above tube). After 2 ml chloroform and 3 ml distilled water were subsequently added, the mixture was vortexed and centrifugated at 10000 g for 10 min. The upper phase was used for glycine betaine content measurement by TSQ Quantum Access MAX triple stage quadrupole mass spectrometer (Thermo, USA) with Hypersil ODS column (5 µm particles size, 4.6×250 mm, USA). The glycinebetaine content was calculated from a standard curve prepared with pure glycinebetaine (Sigma, USA) solutions.

### Measurement of proline content

Leave samples (0.2 g) were homogenized using pestle and mortar with 3ml of 5% (w/v) sulphosalicylic acid and incubated at 100 °C for 10 min. After centrifugation at 13000 g for 10 min, 2 ml glacial acetic acid and 3 ml ninhydrin reagent were added to 2 ml of the supernatant and incubated at 100 ^o^Cfor 40 min. After cooling, 5 ml toluene was added to the mixture and the absorbance at 520 nm of the toluene phase was recorded [10]. The standard curve was plotted according to the proline solution of known concentration.

### Measurement of Na^+^ and Cl^-^ content

The extraction of Na^+^ and Cl^-^ were performed according to Luo et al. [20]. Deionized H_2_O (15 ml) was added to 100 mg of dried plant powder and boiled for 2 h. After centrifugation at 10000 g for 20 min, the supernatant was used for measurement of Cl^-^ content and diluted 100 times with deionized H_2_O for Na^+^ content. The atomic absorption spectrophotometer (PAS-990, PERSEE, China) was used for measurement of Na^+^ content while Cl^-^ content was analyzed with a Cl^-^ electrode (Leici, China). The amount of these three ions was calculated from a standard curve prepared with pure NaCl (for Na^+^ and Cl^-^) solutions.

### Measurements of net Na^+^ and Cl^-^ fluxes

Net fluxes of Na^+^ and Cl^-^ were measured using Non-invasive Micro-test Technique (NMT-YG-100, Younger, USA). The concentration gradients of the target ions were measured by moving the ionselective microelectrode repeatedly between two points close to the plant material. The ion fluxes were calculated based on the Fick’s law of diffusion.

Prepulled and silanized glass micropipettes (Xuyue Sci. and Tech., China) were firstly filled with a backfilling solution (Na^+^: 100 mM NaCl; Cl^-^: 100 mM KCl) to a length of approximately 1 cm from the tip. Then the micropipettes were front filled with selective liquid ion-exchange cocktails (LIXs, Xuyue Sci. and Tech., China). An Ag/AgCl wire electrode holder (Xuyue Sci. and Tech., China) was then inserted in the back of the electrode to make electrical contact with the electrolyte solution. DRIREF-2 (World Precision Instruments) was used as the reference electrode.

Ion-selective electrodes were firstly calibrated before flux measurements using the following solutions: (1) Na^+^: 5 mM, 2 mM, 1 mM (2 mM in measuring solution); (2) K^+^: 1 mM, 0.5 mM, 0.1 mM (0.5 mM in measuring solution); (3) H^+^: pH 5, 6, 7 (6 in measuring solution); (4) Cl^-^: 2 mM, 0.5 mM, 0.25 mM (0.5 mM in measuring solution). Only electrodes with Nernstian slopes >50 mV/decade (< -50 mV/decade for Cl^-^ electrodes) were used.

Root segments with 3 cm apices were immobilized on the bottom of measuring dish, rinsed with deionized water and immediately incubated in the measuring solution to equilibrate for 20 min. The measuring site was 500µm from the root apex, in which a vigorous flux of Na^+^ or Cl^-^ was usually observed. The measuring solutions were as follows: (1) Na^+^: 0.1 mM KCl, 0.1 mM CaCl_2_, 0.1 mM MgCl_2_, 2 mM NaCl, 0.3 mM MES, pH 6.0, adjusted with choline and HCl; (2) Cl^-^: 0.05 mMKCl, 0.05 mM CaCl_2_, 0.05 mM MgCl_2_, 0.25 mM NaCl, pH 6.0, adjusted with choline and H_3_PO_3_. The miro-volts differences were then imported and converted into net ion fluxes using the JCal V3.0 (Xuyue Sci. and Tech., China).

### Measurement of MDA content

The lipid peroxidation level was determined in terms of MDA content by the thiobarbituric acid reaction method [29]. Leaf tissues (0.2 g) were ground under liquid nitrogen and then homogenized in 3.5 ml 200 mM potassium phosphate buffer (pH 7.6) containing 1 mM EDTA, 2% (w/v) PVPP and 1 mM ascorbate. After centrifugation at 4 °C and 13000 g for 10 min, the supernatant was prepared for the measurement. The reaction mixture containing 1 ml of extract supernatant and 4 ml of 0.5% thiobarbituric acid in 20% trichloroacetic acid (TCA) was incubated in water bath at 95 °C for 30 min, and then transferred to ice bath to stop the reaction. After centrifugation at 10000 g for 10 min, the absorbance of supernatant was measured at 532 nm, 600 nm and 450 nm. The concentration of MDA was calculated as: MDA content (µM) = 6.45(A_532_–A_600_)–0.56 A_450_.

### Measurement of total phenolics content

Total phenolics were extracted from 0.1 g of dry leaf powder by homogenization in 8 ml methanol for 2 h. The mixture was centrifuged for 5 min at 10000 g and the supernatant was diluted ten times with methanol before the Folin–Ciocalteu assay [30]. A 0.1 ml aliquot of supernatant was mixed with 0.15 ml 1M Folin–Ciocalteu reagent, 0.15 ml 10% (W/V) Na_2_CO_3_ and 4.6 ml distilled water. The reaction mixture was incubated in water bath at 25 °C for 90 min and the absorbance was measured at 760 nm. Total phenolics content was calculated by comparison with the standard curve obtained with gallic acid.

### Measurement of AsA content

Leaf samples (0.2 g) were ground under liquid nitrogen and homogenized in 5ml 6% (w/v) metaphosphoric acid (MPA) containing 2 mM EDTA and 1% (w/v) PVPP. The homogenate was centrifuged at 13000 g for 20 min and the supernatant was used for measurement by liquid chromatography method [31]. The analysis was performed in a Surveyor Plus high performance liquid chromatography system (Thermo, USA) with a Polaris C18-A column (5.0 µm particles size, 4.6×150 mm). AsA was detected at 243 nm and the content was calculated from a standard curve prepared with pure AsA (Sigma, USA) solutions.

### Measurement of GSH content

GSH was measured according to the method reported by Li et al. [32] with a slight modification. Leave samples (0.2 g) were homogenized using pestle and mortar with 3 ml 5% (w/v) sulphosalicylic acid, the homogenate was then centrifuged at 13000 g for 20 min. A 1 ml aliquot of the supernatant was neutralized with 1.5 ml 0.05 M K-phosphate buffer (pH 7.5) containing 5 mM EDTA, this sample was used for the assay of total glutathione. Another 1 ml aliquot of the supernatant was also neutralized with 1.5 ml 0.05 M K-phosphate buffer (pH 7.5) containing 5 mM EDTA, and then 0.2 ml 2-vinylpyridine was added. The tube was mixed until an emulsion formed and then incubated at 25 °C for 1 h, this sample was used for the assay of oxidized glutathione (GSSG).

The reaction mixture contained: 0.3 ml 2 mM 5,5’-dithiobis-(2-nitrobenzoic acid) (DTNB), 0.5 ml 0.42 mM NADPH, 0.1 ml (1 U) of yeast glutathione reductase (GR, Sigma, USA) (DTNB, NADPH and GR were all dissolved in phosphate buffer (pH 7.5) containing 6 mM EDTA). The reaction was initiated by the addition of 0.1 ml GSH standard or extract. The absorbance change within 2min was recorded and the GSH concentration was proportional to the slope.

### Enzyme extraction and activity assay

Leaf tissues (0. 2g) were ground under liquid nitrogen and then homogenized in 5 ml of 200 mM potassium phosphate buffer (pH 7.8) containing 1 mM EDTA, 2% (w/v) PVPP. After centrifugation at 4 °C and 13000 g for 10 min, the supernatant was prepared for SOD activity assay. By the same procedure, the enzyme extracts for APX activity assay were prepared in 5 ml 200 mM (pH 7.6) potassium phosphate buffer containing 1 mM EDTA, 2% (w/v) PVPP, and 1mM ascorbate.

The assay of SOD activity was based on the method described by Beyer and Fridovich [33]. One unit of enzymatic activity was defined as the amount of enzyme required to bring about a 50% inhibition of the rate of nitro blue tetrazolium (NBT) reduction measured at 560 nm. APX activity was respectively calculated by the rate of AsA consumption and monitored by the change of absorbance at 290 nm [34].

The reaction solution compositions for enzyme activity assay are as follows: (1) SOD: 0.3 ml 13 µM riboflavin, 0.3 ml 130 mM L-methionine (L-Met), 0.3 ml 63 µM NBT and 2.1 ml extract. (2) CAT: 2 ml 15 mM H_2_O_2_ and 1 ml extract. (3) APX: 0.8 ml 0.5m MAsA, 0.1 ml 2m M H_2_O_2_ and 0.1 ml extract (4). DHAR: 0.7 mlpotassium phosphate buffer (pH 7.0), 0.1 ml 50mM reduced glutathione, 0.1 ml 2mM dehydroascorbic acid and 0.1 ml extract.

### Statistical analysis

One-way ANOVA was carried out by using SPSS 16.0 (SPSS Inc., Chicago, IL, USA) for all sets of data. The values presented are the means of all measurements, and comparisons of means were determined through LSD test among the different salt treatments in a cultivar and between the two soybean cultivars under the same salt treatment. Difference was considered significant at *P*< 0.05.

## Results

### Effects of salt stress on chlorophyll content and parameters of gas exchange and chlorophyll fluorescence

**Figure 1:**
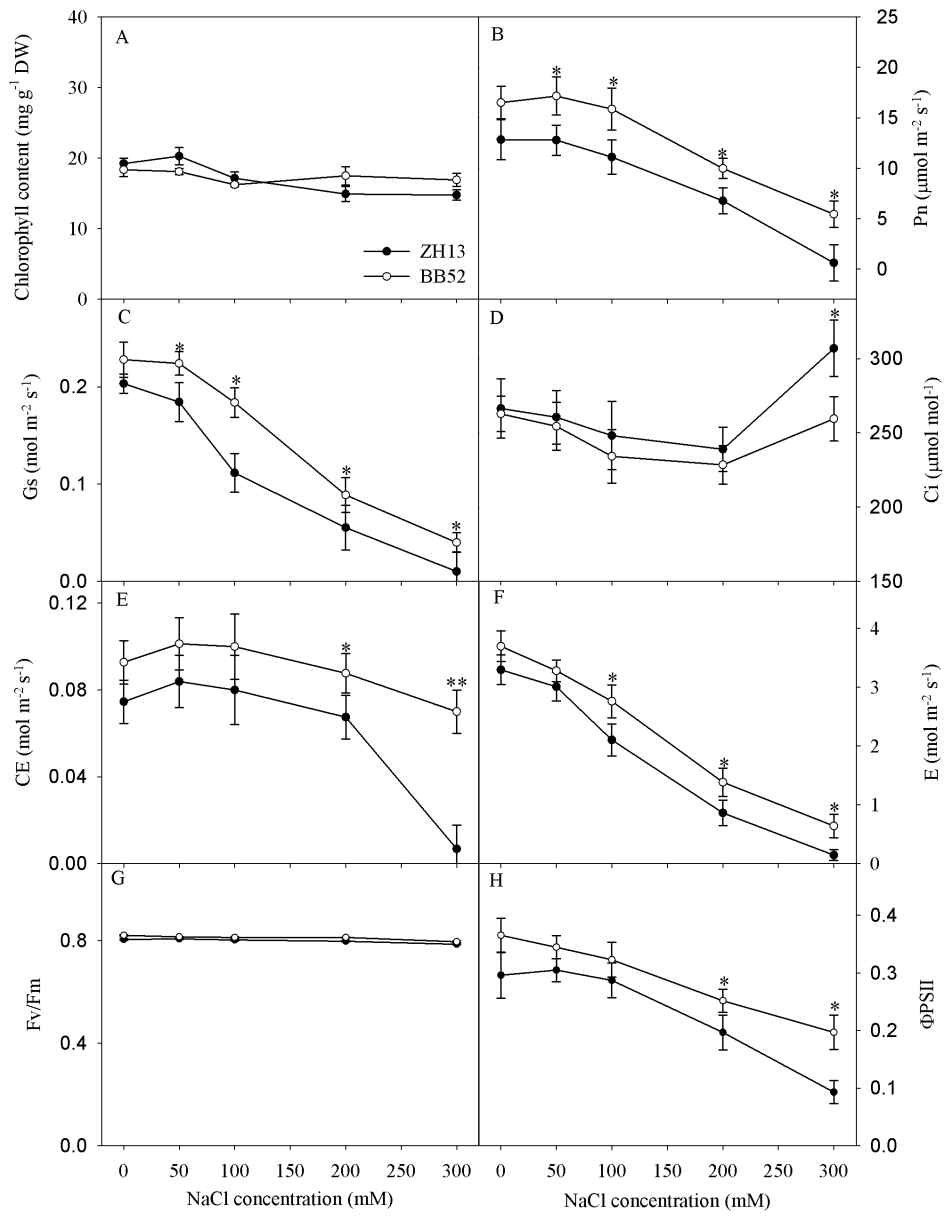
Changes in chlorophyll content (A), photosynthetic rate (Pn, B), stomatal conductance (Gs, C), intercellular CO_2_ concentration (Ci, D), carboxylation efficiency (CE, E), transpiration rate (E, F), maximal photochemical efficiency of PSII (Fv/Fm, G) and actual photochemical efficiency of PSII (ΦPSII, H) under salt stress for 7 days. DW indicates dry weight. Data in the figure indicate mean of five replicates (±SD). Significant difference between *G*. *max* (ZH13) and *G*. *soja* (BB52) is indicated by asterisks: * *P*< 0.05, ** *P* < 0.01.

Chlorophyll content and Fv/Fm were not significantly affected by salt stress in ZH13 and BB52 (Figure 1A-H). Upon salt stress exposure, Pn, Gs, CE, E and ΦPSII decreased with increasing NaCl concentration in ZH13 and BB52, and they were higher in BB52 than in ZH13. In ZH13 and BB52, Ci gradually decreased with increasing NaCl concentration up to 200 mM (Figure 1D). At 300 mM NaCl concentration, Ci increased significantly in ZH13, whereas it increased to the control level in BB52 (Figure 1D).

### Effects of salt stress on rubisco activity and expression of rubisco activase

**Figure 2:**
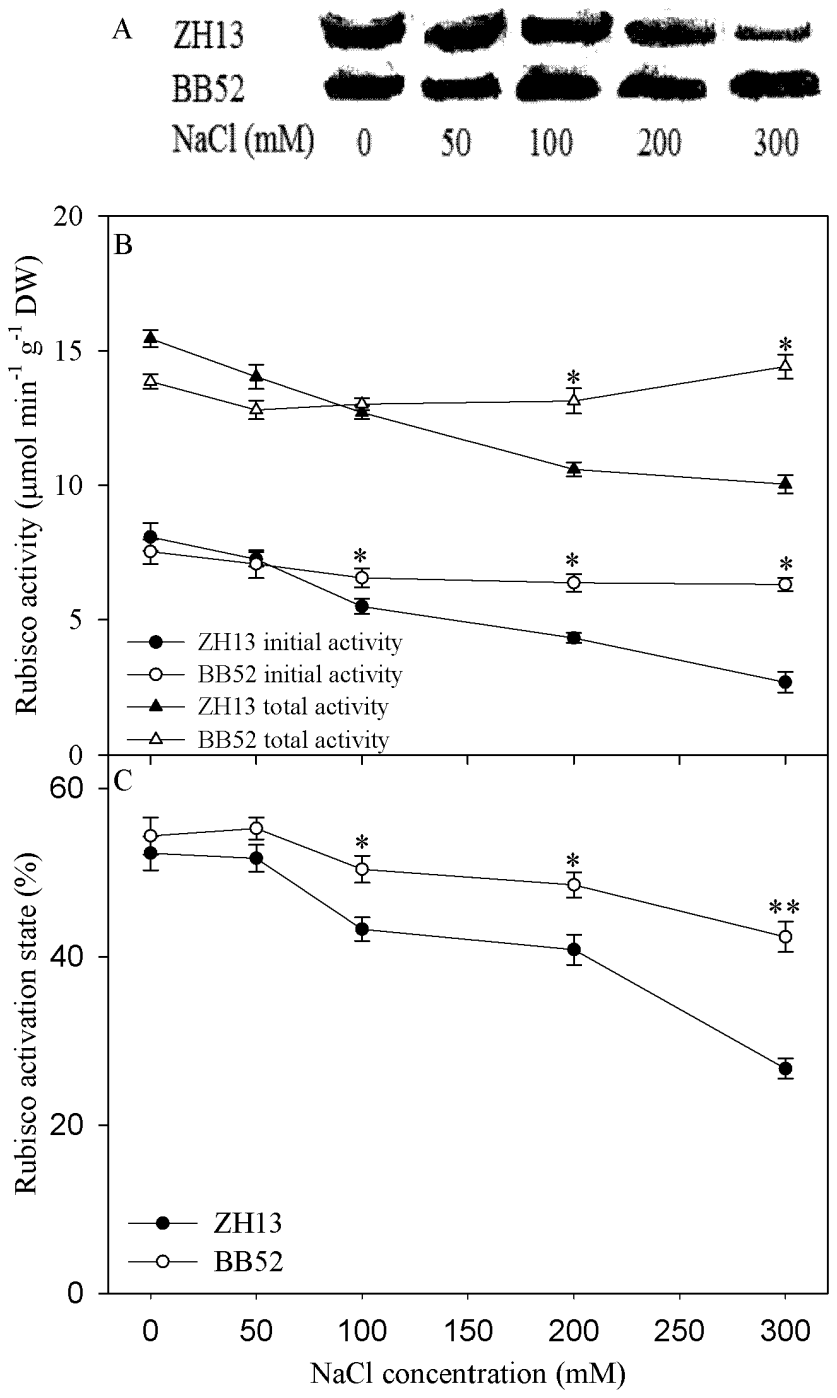
Western-blotting of rubisco activase (A) and changes in rubisco activity (B) and rubisco activation state (C) under salt stress for 7 days. DW indicates dry weight. Data in the figure indicate mean of five replicates (±SD). Significant difference between *G*. *max* (ZH13) and *G*. *soja* (BB52) is indicated by asterisks: * *P*< 0.05, ** *P* < 0.01.

Initial rubisco activity was decreased by salt stress in ZH13 and BB52, and the decrease was greater in ZH13 (*P* < 0.05) (Figure 2A-C). Salt stress had no significant effect on total rubisco activity in BB52, but when NaCl concentration rose to 200 mM, significant decrease in total rubisco activity appeared in ZH13 (*P* < 0.05) (Figure 2B). Rubisco activation state decreased with increasing NaCl concentration and it was significantly higher in BB52 than in ZH13 (Figure 2C). Western blotting showed that rubisco activase content was pronouncedly declined upon 300 mM NaCl exposure, but this enzyme was not affected in BB52 (Figure 2A).

### Effects of salt stress on water potential, relative water content, glycinebetaine content and proline content

**Figure 3:**
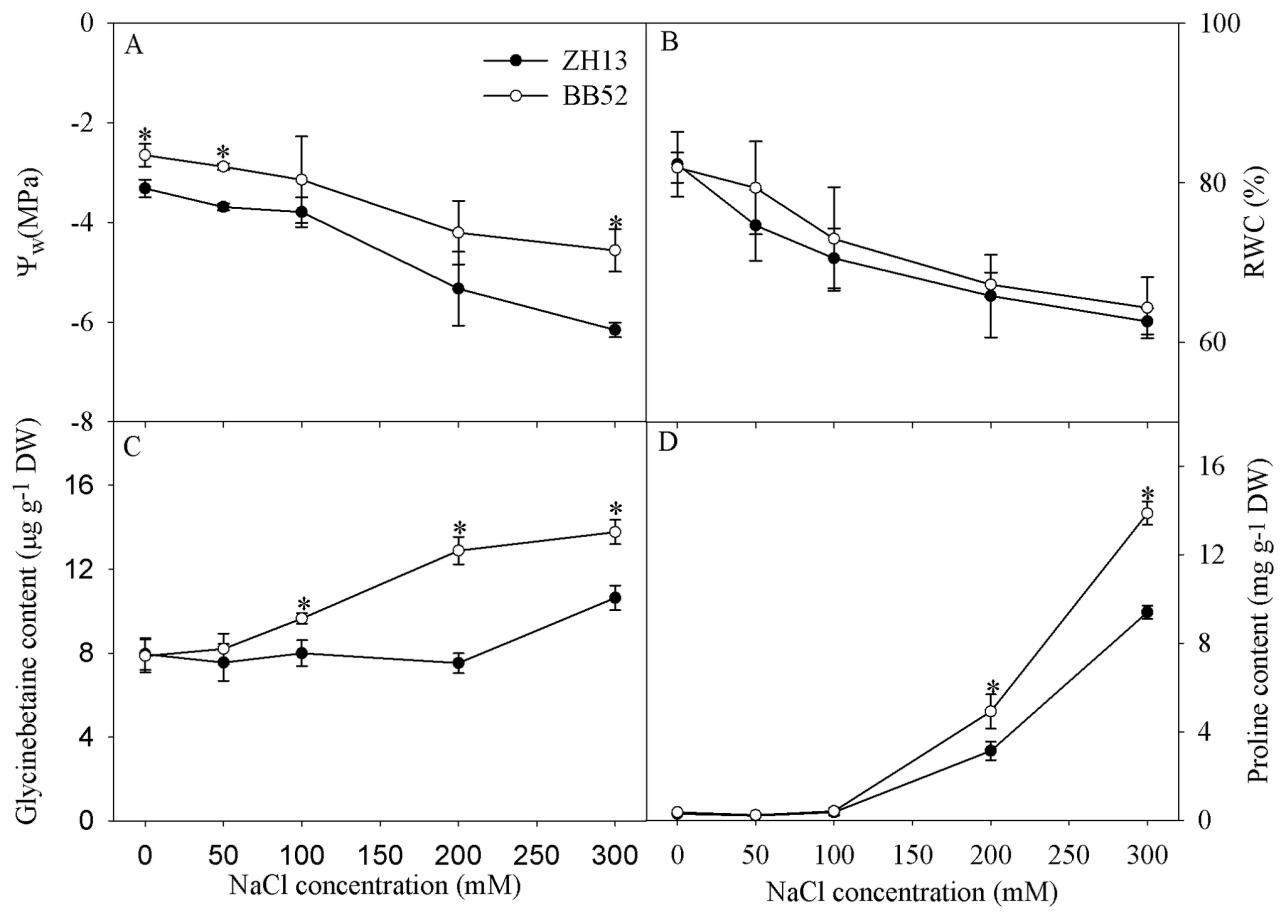
Changes in water potential (ψ_w_, A), relative water content (RWC, B), glycinebetaine (C) and proline (D) contents under salt stress for 7 days. DW indicates dry weight. Data in the figure indicate mean of five replicates (±SD). Significant difference between *G*. *max* (ZH13) and *G*. *soja* (BB52) is indicated by asterisks: * *P* < 0.05, ** *P* < 0.01.

With increasing NaCl concentration, Ψw and RWC decreased in ZH13 and BB52, while glycinebetaine and proline contents increased (Figure 3A-D). Ψw, RWC and contents of glycinebetaine and proline were higher in BB52 than in ZH13 under salt stress, and the difference in RWC between BB52 and ZH13 was not significant (Figure 3C,B).

### Effects of salt stress on Na^+^and Cl^-^ contents and ion flux in the root surface

**Figure 4:**
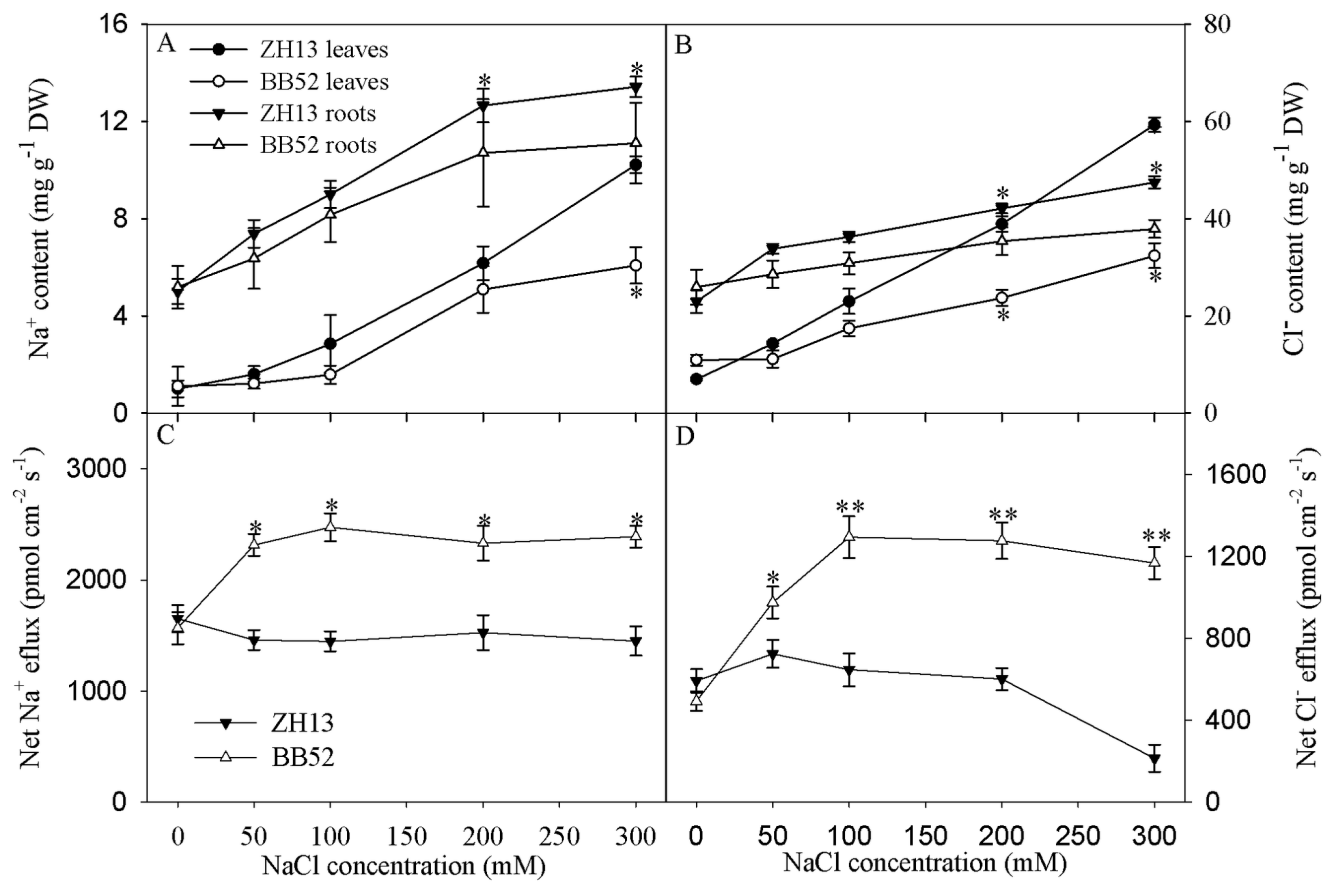
Changes in Na^+^ (A) and Cl^-^ (B) contents in leaves and roots and Na^+^ (C) and Cl^-^(D) fluxes in roots under salt stress for 7 days. DW indicates dry weight. Data in the figure indicate mean of five replicates (±SD). Significant difference between *G*. *max* (ZH13) and *G*. *soja* (BB52) is indicated by asterisks: * *P*< 0.05, ** *P* < 0.01.

In ZH13 and BB52, Na^+^ and Cl^-^ content in leaves and roots increased significantly with increasing NaCl concentration (Figure 4A, B). Under salt stress, BB52 could maintain lower Na^+^ and Cl^-^ contents in leaves and roots. NMT results showed that Na^+^ and Cl^-^ were excluded by roots. Na^+^ efflux of ZH13 was not affected under salt stress, and Cl^-^ efflux of ZH13 remained at a lower level and decreased dramatically under 300 mM NaCl. (Figure 4C, D). Na^+^ and Cl^-^ effluxes of BB52 were significantly increased by 50 mM NaCl, and their effluxes level was not changed significantly with increasing NaCl (Figure 4C, D). Under salt stress, effluxes of Na^+^ and Cl^-^ in BB52 were significantly higher than those in ZH13 (Figure 4C, D). 

### Effects of salt stress on MDA content, total phenolics content and antioxidant system

**Figure 5:**
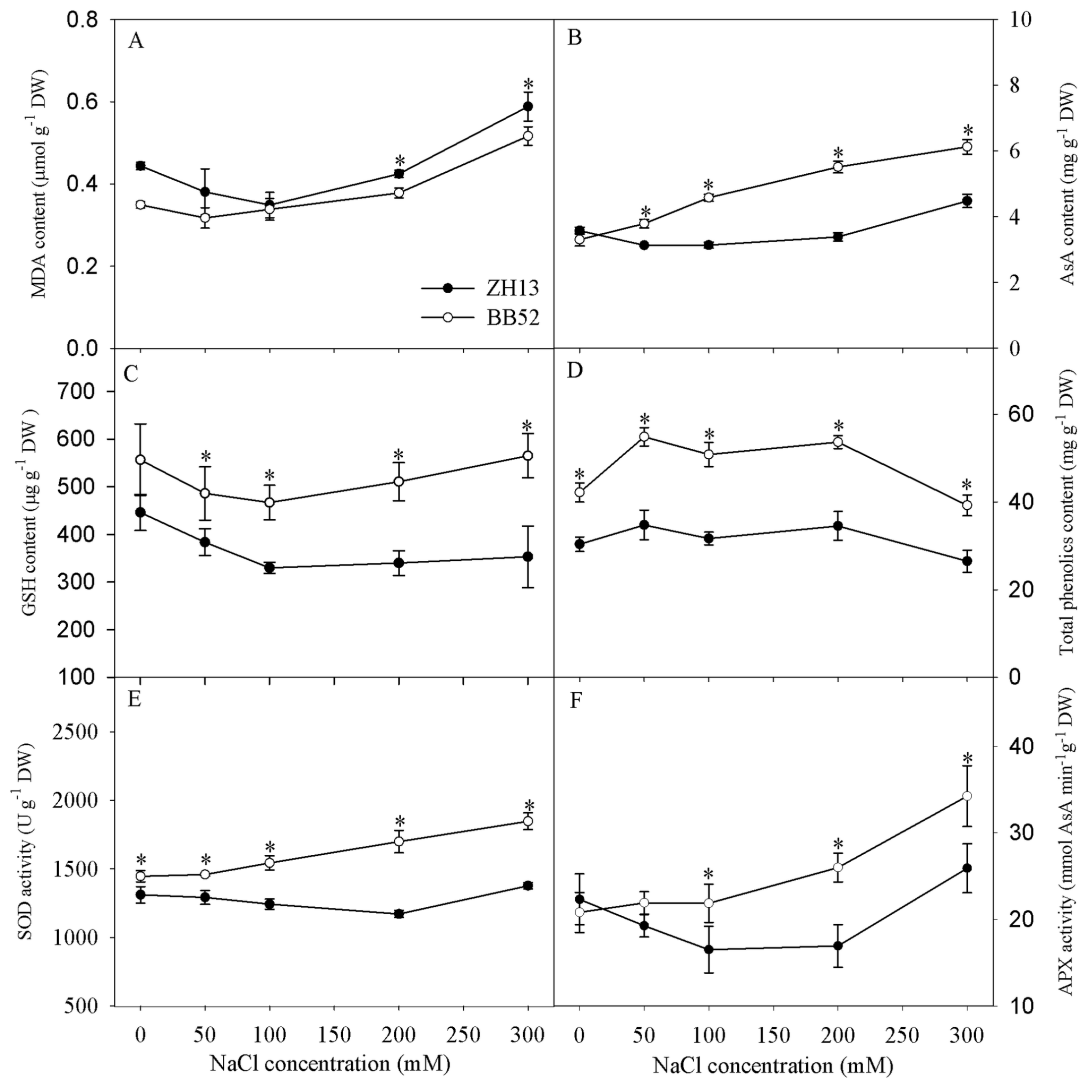
Changes in malondialdehyde (MDA) content (A), ascorbic acid (AsA) content (B), glutathione (GSH) content(C), total phenolics content (D) and activities of superoxide dismutase (SOD, E) and ascorbate peroxidase (APX, F) under salt stress for 7 days. DW indicates dry weight. Data in the figure indicate mean of five replicates (±SD). Significant difference between *G*. *max* (ZH13) and *G*. *soja* (BB52) is indicated by asterisks: * *P*< 0.05, ** *P* < 0.01.

When NaCl treatment was over 100 mM, MDA content increased significantly in the leaves of ZH13. Salt-induced increase in MDA content also appeared in leaves of BB52, but the increase was lower than that in ZH13 (Figure 5A-F). Under salt stress, BB52 maintained significantly higher total phenolics, GSH and AsA contents in the leaves in contrast to ZH13 (Figure 5B-D). Under salt stress, SOD and APX activities were stimulated in BB52 and ZH13 leaves, and they were significantly higher in BB52 than in ZH13 (Figure 5E, F).

## Discussion

Salt-induced decrease of Pn in BB52 and ZH13 indicated that photosynthesis was negatively affected by salt stress (Figure 1B). Under salt stress, Pn was significantly higher in BB52 than in ZH13, suggesting the stronger salt tolerance of photosynthesis in BB52 (Figure 1B). According to the theory of Farquhar and Sharkey [35], salt-induced decrease in Pn was mainly due to stomatal limitation in BB52 and ZH13 before NaCl concentration reached 300 mM, as G_s_ and Ci decreased simultaneously (Figure 1C, D). The salt-induced stomatal limitation of Pn, as a common result, has been observed in other crops such as sorghum, rice and maize [10,11,36], and the decrease in Gs also can reduce water loss from leaf transpiration (Figure 1F). Under 300 mM NaCl, increase in Ci along with decrease in Gs appeared in BB52 and ZH13 (Figure 1D), and thus, the main limitation of Pn derived from non-stomatal factor. In our opinion, decreased Pn resulted from the depressed carboxylation process at 300 mM NaCl, as significant decrease in CE appeared (Figure 1E). ΦPSII also was significantly decreased at 300 mM NaCl, indicating photosynthetic electron transport rate was declined, but we did not believe that the decreased ΦPSII was the underlying cause for the decreased Pn. Fv/Fm is a classic parameter reflecting PSII stability. Unlike Fv/Fm, ΦPSII reflects PSII photochemical efficiency after photosynthetic initiation. Fv/Fm and chlorophyll content was not affected by salt stress (Figure 1 A, G), and therefore, the decreased ΦPSII did not result from PSII damage but might be due to the increased non-photochemical quenching or the feedback inhibition of declined CO_2_ fixation. Oppositely, Mehta et al. [9] and Kalaji et al. [12] reported that salt stress led to PSII damage in wheat and barley. We believed that detached leaves were used in their studies, and salt tolerance of PSII cannot be maintained. Clearly, CE was significantly higher in BB52 than in ZH13 under 300 mM NaCl, indicating the stronger salt tolerance of CO_2_ fixation process in BB52. Rubisco is responsible for CO_2_ fixation in C3 plants, but it is still an open debate about the salt effects on rubisco. The existing studies showed that salt stress inhibited CO_2_ carboxylation of rubisco by decreasing rubisco content or inhibit rubisco activation [8,11]. In this study, rubisco content might not be affected by salt stress, since total activity of rubisco did not changed (Figure 2B), whereas rubisco activation was inhibited indicated by the declined rubisco initial activity and activation state (Figure 2B, C). Rubisco activase plays an important role in rubisco activation, and heat sensitivity of this enzyme has been widely demonstrated [37,38]. As illustrated by Western-blotting, rubisco activase content was greatly decreased in ZH13 at 300 mM NaCl, while negative effect cannot be discerned in BB52 (Figure 2A). Thus, stronger salt tolerance of rubisco activase could explain the higher photosynthetic activity in BB52 under severe salt stress.

High salt resistance of BB52 can be reflected in terms of osmotic regulation and antioxidant system. Water is a key factor for crop growth as well as plant photosynthesis. Under salt stress, Ψw and RWC were decreased in BB52 and ZH13 leaves, but they were relatively higher in BB52 (Figure 3A, B), indicating that BB52 had higher osmotic regulation capacity to maintain relatively proper water status. The major role of proline and glycinebetaine is accepted as the osmolyte in plants grown in saline soil, and they are proposed as the potential indicators for salt tolerance in plants [39]. In this study, higher accumulation of proline and glycinebetaine was discerned in BB52 leaves upon NaCl exposure in contrast to ZH13 (Figure 3C, D), helping to better alleviated the osmotic pressure. Salt-induced oxidative attack can be mitigated by the positive response of antioxidant system, and antioxidant capacity is usually used as the criterion for discriminating salt tolerant crop cultivar [17,40]. In this study, oxidative damage was less in BB52 than in ZH13, as illuminated by the lower MDA content in the leaves (Figure 5A). Correspondingly, higher activities of antioxidant enzymes such as SOD and APX were detected in BB52 leaves in addition to its more accumulation of antioxidants such as AsA, GSH and total phenolics (Figure 5B, C, D). Thus, BB52 possesses high salt tolerance partly by virtue of antioxidant capacity as well.

Avoiding accumulation of toxic ions of Na^+^ and Cl^-^ is an important way to resist salt stress [1]. Less Na^+^ and Cl^-^ contents are often recorded in the tissues of salt tolerant crops cultivar compared with salt sensitive cultivar [41]. Under salt stress, Na^+^ and Cl^-^ contents were less in leaves and roots of BB52 than in ZH13 (Figure 4A, B), and this result was attributed to the higher excluding flux for Na^+^ and Cl^-^ in BB52 roots (Figure 4C, D). Consistently, Sun et al. [19] also proved that salt-tolerant poplar cultivar had greater ability to exclude Na^+^ and Cl^-^. In particular, Zhang et al. [21] have revealed the higher efflux of Na^+^ and Cl^-^ in BB52 roots under 140 mM NaCl by using NMT. However, our study further showed that the efflux of Na^+^ and Cl^-^ was enhanced under 50 mM NaCl, and the efflux was not changed with increasing NaCl concentration (Figure 4C, D). Thus, we infer that BB52 resistance to severe salt stress cannot only depend on the root exclusion of Na^+^ and Cl^-^.

In conclusion, BB52 can maintain higher photosynthetic efficiency under salt stress, and the higher salt resistance in BB52 depends on the coordination of antioxidant system, osmotic regulation and toxic ion exclusion.

## References

[1] Munns R, Tester M (2008) Mechanisms of salinity tolerance. Annu Rev Plant Biol 59: 651-681. doi:10.1146/annurev.arplant.59.032607.092911. PubMed: 18444910. 18444910

[2] Rozema J, Flowers T (2008) Crops for a Salinized World. Science 322: 1478-1480. doi:10.1126/science.1168572. PubMed: 19056965. 19056965

[3] Munns R, James RA, Läuchli A (2006) Approaches to increasing the salt tolerance of wheat and other cereals. J Exp Bot 57: 1025-1043. doi:10.1093/jxb/erj100. PubMed: 16510517. 16510517

[4] Flowers TJ, Colmer TD (2008) Salinity tolerance in halophytes. New Phytol 179: 945-963. doi:10.1111/j.1469-8137.2008.02531.x. PubMed: 18565144. 18565144

[5] Yan K, Shao H, Shao C, Chen P, Zhao S et al. (2013) Physiological adaptive mechanisms of plants grown in saline soil and implications for sustainable saline agriculture in coastal zone. Acta Physiol Plant 35: 2867-2878. doi:10.1007/s11738-013-1325-7.

[6] Ashraf M, Foolad MR (2007) Roles of glycine betaine and proline in improving plant abiotic stress resistance. Environ Exp Bot 59: 206-216. doi:10.1016/j.envexpbot.2005.12.006.

[7] Loreto F, Centritto M, Chartzoulakis K (2003) Photosynthetic limitations in olive cultivars with different sensitivity to salt stress. Plant Cell Environ 26: 595-601. doi:10.1046/j.1365-3040.2003.00994.x.

[8] Lu KX, Cao BH, Feng XP, He Y, Jiang DA (2009) Photosynthetic response of salt-tolerant and sensitive soybean varieties. Photosynthetica 47: 381-387. doi:10.1007/s11099-009-0059-7.

[9] Mehta P, Jajoo A, Mathur S, Bharti S (2010) Chlorophyll a fluorescence study revealing effects of high salt stress on Photosystem II in wheat leaves. Plant Physiol Biochem 48: 16-20. doi:10.1016/j.plaphy.2009.10.006. PubMed: 19932973. 19932973

[10] Yan K, Chen P, Shao H, Zhao S, Zhang L et al. (2012) Responses of photosynthesis and photosystem II to higher temperature and salt stress in Sorghum. J Agron Crop Sci 198: 218-226. doi:10.1111/j.1439-037X.2011.00498.x.

[11] Feng LL, Han YJ, Liu G, An BG, Yang J et al. (2007) Overexpression of sedoheptulose-1,7-bisphosphatase enhances photosynthesis and growth under salt stress in transgenic rice plants. Funct Plant Biol 34: 822-834. doi:10.1071/FP07074. 32689410

[12] Kalaji HM, Govindjee, Bosa K, Koscielniak J, Zuk-Golaszewska K (2011) Effects of salt stress on photosystem II efficiency and CO_2_ assimilation of two Syrian barley landraces. Environ Exp Bot 73: 64-72. doi:10.1016/j.envexpbot.2010.10.009.

[13] Netondo GW, Onyango JC, Beck E (2004) Sorghum and salinity: II. Gas exchange and chlorophyll fluorescence of sorghum under salt stress. Crop Sci 44: 806-811. doi:10.2135/cropsci2004.0806.

[14] Lu CM, Qiu NW, Wang BS, Zhang JH (2003) Salinity treatment shows no effects on photosystem II photochemistry, but increases the resistance of photosystem II to heat stress in halophyte *Suaeda* *salsa* . J Exp Bot 54: 851-860. doi:10.1093/jxb/erg080. PubMed: 12554728. 12554728

[15] Tarchoune I, Degl'Innocenti E, Kaddour R, Guidi L, Lachaal M et al. (2012) Effects of NaCl or Na2SO_4_ salinity on plant growth, ion content and photosynthetic activity in *Ocimum* *basilicum* L. Acta Physiol Plant 34: 607-615. doi:10.1007/s11738-011-0861-2.

[16] Turkan I, Demiral T (2009) Recent developments in understanding salinity tolerance. Environ Exp Bot 67: 2-9. doi:10.1016/j.envexpbot.2009.05.008.

[17] Neto ADD, Prisco JT, Eneas J, de Abreu CEB, Gomes E (2006) Effect of salt stress on antioxidative enzymes and lipid peroxidation in leaves and roots of salt-tolerant and salt-sensitive maize genotypes. Environ Exp Bot 56: 87-94. doi:10.1016/j.envexpbot.2005.01.008.

[18] Sairam RK, Srivastava GC, Agarwal S, Meena RC (2005) Differences in antioxidant activity in response to salinity stress in tolerant and susceptible wheat genotypes. Biol Plant 49: 85-91. doi:10.1007/s10535-005-5091-2.

[19] Sun J, Chen SL, Dai SX, Wang RG, Li NY et al. (2009) NaCl-induced alternations of cellular and tissue ion fluxes in roots of salt-resistant and salt-sensitive poplar species. Plant Physiol 149: 1141-1153. PubMed: 19028881. 19028881 10.1104/pp.108.129494 PMC2633858

[20] Luo QY, Yu BJ, Liu YL (2005) Differential sensitivity to chloride and sodium ions in seedlings of *Glycine* *max* and *G.* *soja* under NaCl stress. J Plant Physiol 162: 1003-1012. doi:10.1016/j.jplph.2004.11.008. PubMed: 16173461. 16173461

[21] Zhang XK, Zhou QH, Cao JH, Yu BJ (2011) Differential Cl-/salt tolerance and NaCl-induced alternations of tissue and cellular ion fluxes in *Glycine* *max*, *Glycine* *soja* and their hybrid seedlings. J Agron Crop Sci 197: 329-339.

[22] Lee JD, Yu JK, Hwang YH, Blake S, So YS, et al. (2008) Genetic diversity of wild soybean (*Glycine* *soja* Sieb. and Zucc.) accessions from South Korea and other countries. Crop Sci 48: 606-616.

[23] Hyten DL, Song QJ, Zhu YL, Choi IY, Nelson RL et al. (2006) Impacts of genetic bottlenecks on soybean genome diversity. Proc Natl Acad Sci U S A 103: 16666-16671. doi:10.1073/pnas.0604379103. PubMed: 17068128. 17068128 PMC1624862

[24] Maxwell K, Johnson GN (2000) Chlorophyll fluorescence - a practical guide. J Exp Bot 51: 659-668. doi:10.1093/jexbot/51.345.659. PubMed: 10938857. 10938857

[25] Chen HX, Li WJ, An SZ, Gao HY (2004) Characterization of PS II photochemistry and thermostability in salt-treated *Rumex* leaves. J Plant Physiol 161: 257-264. doi:10.1078/0176-1617-01231. PubMed: 15077623. 15077623

[26] Hartmut K (1983) Determinations of total carotenoids and chlorophylls b of leaf extracts in different solvents. Analysis (Peach, K & Tracey, MV, eds) 4: 142-196

[27] Wang M, Shi S, Lin F, Hao ZQ, Jiang P et al. (2012) Effects of soil water and nitrogen on growth and photosynthetic response of Manchurian Ash (*Fraxinus* *mandshurica*) seedlings in Northeastern China. PLOS ONE 7: e30754. doi:10.1371/journal.pone.0030754. PubMed: 22347401. 22347401 PMC3275608

[28] Bradford MM (1976) A rapid and sensitive method for the quantitation of microgram quantities of protein utilizing the principle of protein-dye binding. Anal Biochem 72: 248-254. doi:10.1016/0003-2697(76)90527-3. PubMed: 942051. 942051

[29] Heath RL, Packer L (1968) Photoperoxidation in isolated chloroplasts: I. Kinetics and stoichiometry of fatty acid peroxidation. Arch Biochem Biophys 125: 189-198. doi:10.1016/0003-9861(68)90654-1. PubMed: 5655425. 5655425

[30] Singleton VL, Orthofer R, Lamuela-Raventos RM (1999) Analysis of total phenols and other oxidation substrates and antioxidants by means of Folin-Ciocalteu reagent. Methods Enzymol 299: 152-178.

[31] Yan K, Chen W, He XY, Zhang GY, Xu S et al. (2010) Responses of photosynthesis, lipid peroxidation and antioxidant system in leaves of *Quercus* *mongolica* to elevated O_3_ . Environ Exp Bot 69: 198-204. doi:10.1016/j.envexpbot.2010.03.008.

[32] Li PM, Castagnoli S, Cheng LL (2008) Red ‘Anjou’ pear has a higher photoprotective capacity than green ‘Anjou’. Physiol Plant 134: 486-498. doi:10.1111/j.1399-3054.2008.01155.x. PubMed: 18715235. 18715235

[33] Beyer WF, Fridovich I (1987) Assaying for superoxide-dismutase activity - Some large consequences of minor changes in Conditions. Anal Biochem 161: 559-566. doi:10.1016/0003-2697(87)90489-1. PubMed: 3034103. 3034103

[34] Krivosheeva A, Tao DL, Ottander C, Wingsle G, Dube SL et al. (1996) Cold acclimation and photoinhibition of photosynthesis in Scots pine. Planta 200: 296-305.

[35] Farquhar GD, Sharkey TD (1982) Stomatal conductance and photosynthesis. Annu Rev Plant Physiology 33: 317-345. doi:10.1146/annurev.pp.33.060182.001533.

[36] Yang XH, Lu CM (2005) Photosynthesis is improved by exogenous glycinebetaine in salt-stressed maize plants. Physiol Plant 124: 343-352. doi:10.1111/j.1399-3054.2005.00518.x.

[37] Salvucci ME, Crafts-Brandner SJ (2004) Relationship between the heat tolerance of photosynthesis and the thermal stability of rubisco activase in plants from contrasting thermal environments. Plant Physiol 134: 1460-1470. doi:10.1104/pp.103.038323. PubMed: 15084731. 15084731 PMC419822

[38] Scafaro AP, Yamori W, Carmo-Silva AE, Salvucci ME, von Caemmerer S et al. (2012) Rubisco activity is associated with photosynthetic thermotolerance in a wild rice (*Oryza* *meridionalis*). Physiol Plant 146: 99-109. doi:10.1111/j.1399-3054.2012.01597.x. PubMed: 22324885. 22324885

[39] Ashraf M, Harris PJC (2004) Potential biochemical indicators of salinity tolerance in plants. Plant Sci 166: 3-16. doi:10.1016/j.plantsci.2003.10.024.

[40] Masood A, Shah NA, Zeeshan M, Abraham G (2006) Differential response of antioxidant enzymes to salinity stress in two varieties of *Azolla* (*Azolla* *pinnata* and *Azolla* *filiculoides*). Environ Exp Bot 58: 216-222. doi:10.1016/j.envexpbot.2005.08.002.

[41] Hussain S, Luro F, Costantino G, Ollitrault P, Morillon R (2012) Physiological analysis of salt stress behaviour of citrus species and genera: Low chloride accumulation as an indicator of salt tolerance. S Afr J Bot 81: 103-112. doi:10.1016/j.sajb.2012.06.004.

